# The Periosteum–Patellar Tendon–Bone Autograft for Anatomic, Single-Bundle Anterior Cruciate Ligament Reconstruction With Press-Fit Tibial Fixation

**DOI:** 10.1016/j.eats.2024.103021

**Published:** 2024-04-30

**Authors:** Yavuz Kocabey, Ahmet Fırat, Ahmet Yıldırım, Ahmet Emre Paksoy, Kerim Öner, Enejd Veizi

**Affiliations:** a Yavuz Kocabey Private Clinic, Istanbul, Turkey; b Department of Orthopedics and Traumatology, Ankara City Hospital, Ankara, Turkey; c Department of Orthopedics and Traumatology, Konya City Hospital, Konya, Turkey; d Department of Orthopedics and Traumatology, Atatürk University, Erzurum, Turkey; e Department of Orthopedics and Traumatology, Karadeniz Technical University, Trabzon, Turkey; f Ankara Yıldırım Beyazıt University, Ankara, Turkey

## Abstract

Optimal graft choice and fixation technique are still ongoing topics of debate for primary and revision anterior cruciate ligament reconstruction. Interference screws are frequently used as graft fixation devices but can sometimes lead to tunnel widening, cyst formation, chronic inflammation, screw breakage, and persistent pain. Tibial tunnel widening is of special concern because it is often associated with graft failure. This technical note introduces a graft technique with a periosteum–patellar tendon–bone autograft and a press-fit tibial fixation approach that could be a viable option for secure anterior cruciate ligament reconstruction while offering the possibility of a quicker postoperative recovery, less pain, and a quicker return to everyday activities.

Anterior cruciate ligament (ACL) reconstruction (ACLR) surgery is one of the most frequently performed orthopaedic procedures worldwide, and still, the optimal graft choice and fixation technique are ongoing topics of debate.^1^^,^^2^ Biocompatible and, nowadays, biodegradable interference screws are the most frequently used graft fixation modality, and although they provide adequate stabilization and serve as an osteoconductive means for complete or partial tunnel ossification, screw-related complications are still frequent.^3^^,^^4^

Cyst formation, chronic inflammation, screw breakage, and persistent pain are some of the most frequent interference screw–related complications.^5^ Several techniques, such as all-inside ACLR, have been described to circumvent these complications, but they are often demanding and costly.^6^ In recent years, an increasing interest has been shown in press-fit techniques to secure the autograft.^7^^,^^8^ Although not necessarily new, these techniques offer full and quick ossification of both tunnels or one of the tunnels and have been reported to be biomechanically robust while clinically reliable.^8^

After an ACLR procedure, the tibial tunnel is more prone to widening, not only because of the bony morphology surrounding the graft but also because of the forces it applies during knee flexion and pivoting movements.^9^ Historically, tibial tunnel defects have been more challenging to manage during revision surgery than femoral tunnel defects, which most often happen to be misplaced. The purpose of this study is to describe the autograft option of a previously described technique^10^^,^^11^ for primary and revision ACLR surgery.

## Surgical Technique

### Patient Position and Initial Preparation

The patient is placed supine on the surgical table (Video 1), the leg holder is removed from beneath the index extremity, and the knee is left floating in 90° of flexion. A tourniquet is routinely used. After proper skin preparation and draping, a diagnostic arthroscopy is performed, confirming the ACL rupture. Graft harvesting follows.

### Graft Harvesting and Preparation

**Fig 1:**
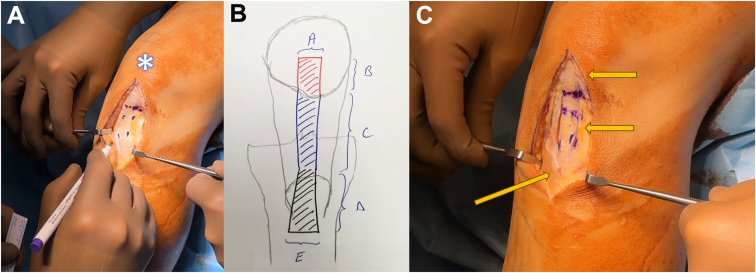
The left knee of a supine-positioned patient is shown. (A, B) A longitudinal midline incision is performed, starting from the center of the patella down to the tibial tubercle. The asterisk marks the superior pole of the patella. The paratenon is carefully dissected, split with a midline incision, and retracted laterally. A ruler is then used to mark a tendon thickness of 10 mm (A) from the middle of the patellar tendon (C). Superiorly, the patellar periosteum is marked with a length of 30 to 40 mm (B) and a width of 10 mm (A). Inferiorly, the tibial tubercle is marked in a trapezoidal shape with a length of approximately 35 mm (D), a narrow superior base of 10 mm (A) and a wider inferior base of 12 mm (E). (C) The marked patellar tendon is released, with each arrow indicating portion B, portion C, or portion D.

**Fig 2:**
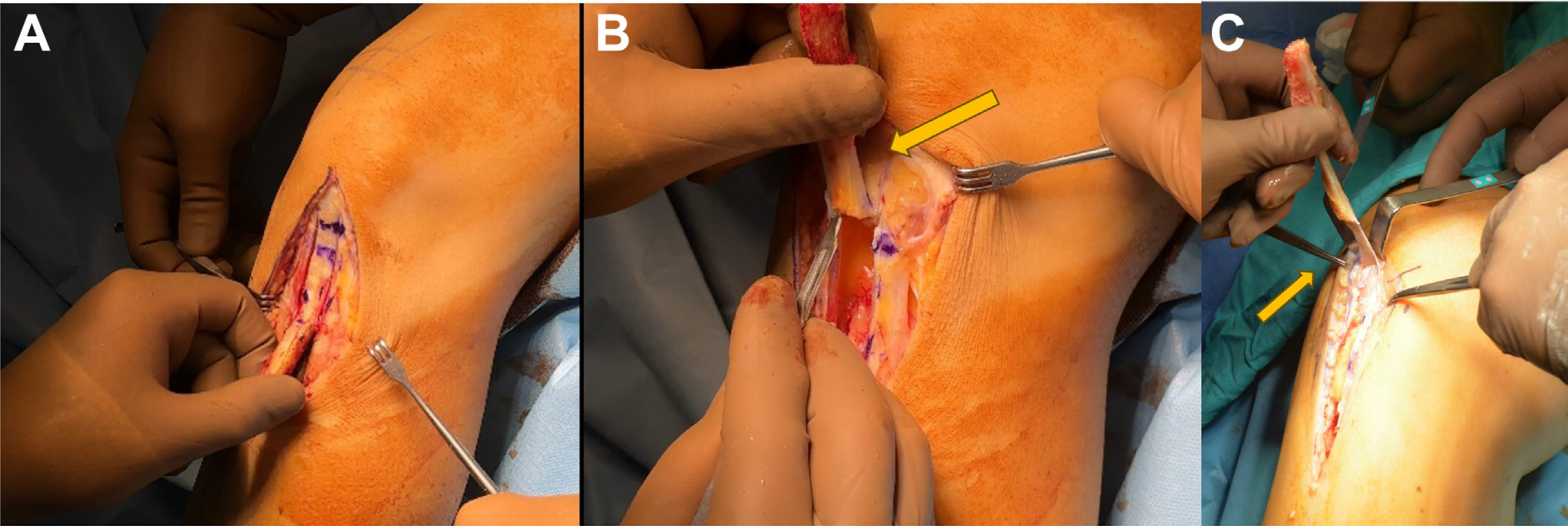
The left knee of a supine-positioned patient is shown. (A) After the release of the patellar tendon, the marked bony end of the tibial tubercle is first cut with an oscillating saw blade and then released with a mallet. (B) The patellar tendon is freed from the surrounding fat pad and elevated (arrow). (C) The marked section of the patellar periosteum is released up to the measured point and detached (arrow).

**Fig 3:**
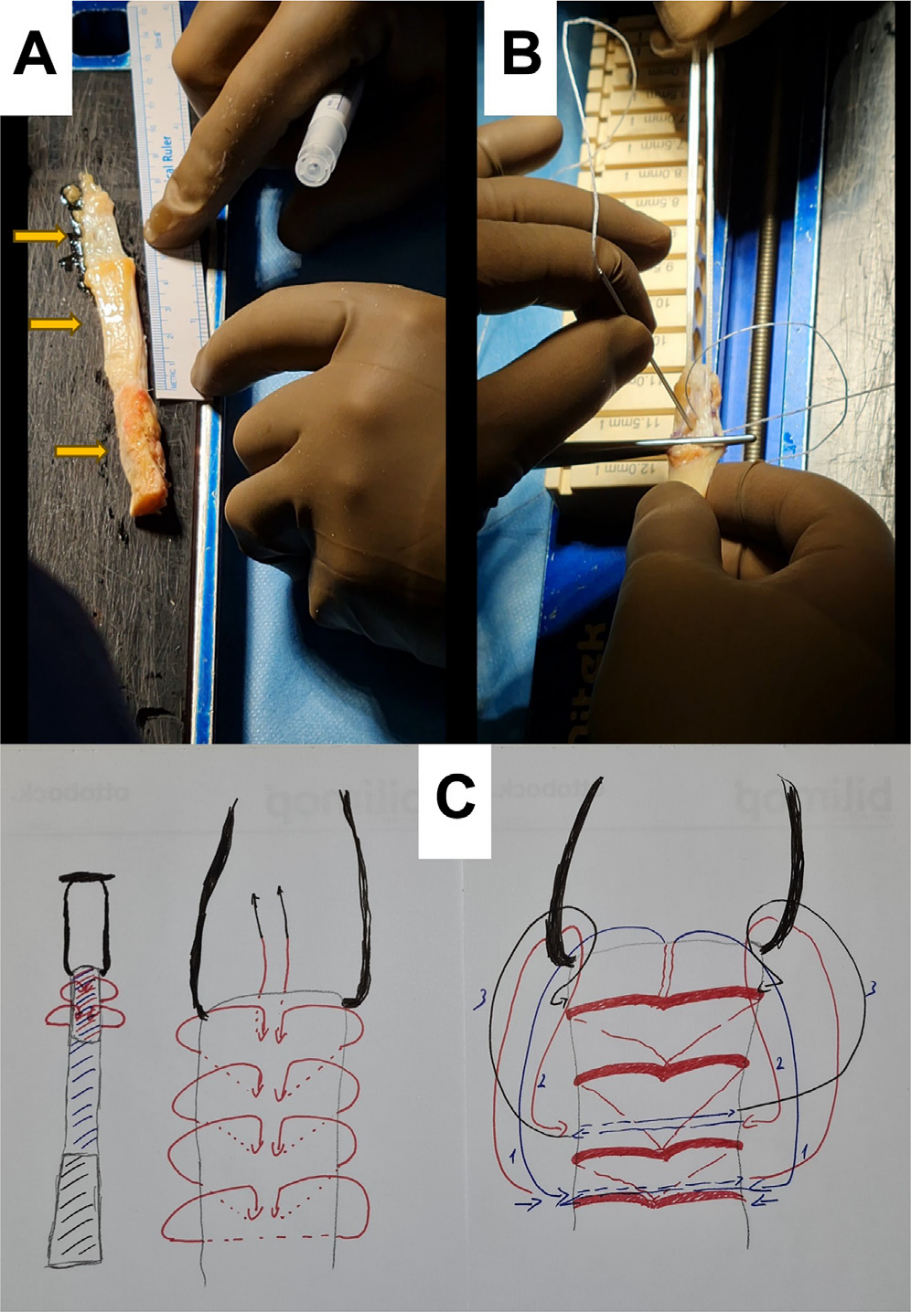
The released graft is remeasured so that the whole tendon length is approximately 45 mm. (A) A mark is placed 20 mm distal to the proximal pole of the graft end to designate the graft amount destined for the femoral tunnel. The arrows, from top to bottom, indicate the periosteal section, patellar tendon, and bony plug. (B) Starting from the 20-mm mark and working our way proximally, the tendon, the periosteum, and the loop of the adjustable fixation device are fixed with a double-needled reinforced loop suture in a whipstitch fashion. After 3 or 4 passages, the needles meet at the distal end of the patellar tendon graft and are shuttled first through the loop of the adjustable fixation device and then twice from lateral to medial through the graft. (C) The suture ends are knotted.

A longitudinal incision is performed, starting from the center of the patella down to the tibial tubercle (Fig 1). The paratenon is dissected, split with a midline incision, and retracted. A ruler is then used to mark a tendon thickness of 10 mm from the middle of the patellar tendon. Superiorly, the patellar periosteum is marked with a length of 30 to 40 mm and a width of 10 mm. Inferiorly, the tibial tubercle is marked in a trapezoidal shape with a length of approximately 35 mm, a narrow superior base of 10 mm, and a wider inferior base of 12 mm. The patellar tendon part of the graft is then released with a knife. The tibial tubercle is cut with an oscillating saw blade (Fig 2). After extraction with a mallet, the construct is pulled superiorly, and the marked section of the periosteum is carefully dissected and released. The graft is obtained and is cleaned on a separate table. The bony part of the graft is placed into a graft tensioner. The loop of the adjustable fixation device is then brought in, and the periosteum is flipped over the loop and is clamped with the proximal part of the patellar tendon (Fig 3). The whole tendon length is adjusted to approximately 45 mm. A mark is placed 20 mm distal to the proximal pole of the graft end to designate the graft amount destined for the femoral tunnel. Starting from the 20-mm mark and working our way proximally, the tendon, the periosteum, and the loop of the adjustable fixation device are fixed with a double-needled reinforced loop suture (Piex Strong Braided Loop Suture Double Needle, Doratek, Ankara, Turkey) in a whipstitch fashion. After 3 or 4 passages, the needles meet at the distal end of the patellar tendon graft and are shuttled first through the loop of the adjustable fixation device and then twice from lateral to medial through the graft. The suture ends are knotted.

Subsequently, the bone block is shaped for the proximal end to pass through the 10-mm hole of the graft sizer and for the distal part to pass through the 12-mm hole. Generally, a femoral tunnel of 8 mm is enough to accommodate the superior part of the graft, and a tunnel of 20 mm is drilled at the 2-o’clock position (or 10-o’clock position, depending on the knee) over the original footprint of the anteromedial bundle of the ACL. The tibial tunnel is then drilled with a planned length of 35 mm and a width of 8 mm. The distal-most end of the tunnel is additionally drilled with a 10-mm reamer for a distance of 10 mm. The graft is then inserted through the tibial tunnel.

### Graft Incorporation and Fine-Tuning

The adjustable fixation device (Knitrope Upper Side Pull Out, Doratek, Ankara, Turkey) is shuttled into the femoral tunnel first and is flipped just above the cortex. The tendon end of the graft is then pulled with the adjustable loop system until the bony end of the graft enters the tibial tunnel distally. A small mallet is used to advance the bony end of the graft while, at the same time, the femoral end is continuously tensioned up to the marked level on the graft. Once adequate tension has been achieved intra-articularly, the residual bony protrusion of the tibial end of the graft is trimmed with the oscillating saw blade. Passive range-of-motion cycles are performed, and the intra-articular tension is rechecked (Table 1). The portals and the longitudinal wound are closed in routine fashion with interrupted sutures.

**Table 1:** Advantages and Disadvantages of Periosteum–Patellar Tendon–Bone Autograft

Advantages
Requires no interference screw for further fixation
Promotes faster bone-to-bone healing
Offers full and intact tibial tunnel in case of future revision
Can be used as single-stage procedure during revision ACLR surgery
Disadvantages
Creates larger defect on tibial tubercle
Requires meticulous intraoperative planning to achieve adequate fixation
Has inherent risk of tibial plateau fracture

ACLR, anterior cruciate ligament reconstruction.

### Postoperative Rehabilitation

On the first day after surgery, patients are started on a weight-bearing-as-tolerated protocol with full passive range of motion and quadriceps strengthening exercises. Full weight bearing is aimed for as soon as patients feel comfortable with it.

## Discussion

The periosteum–patellar tendon–bone autograft aims to provide quicker osseointegration in the tibial tunnel with a biomechanically robust reconstruction and secure femoral fixation. Further clinical studies are already under way to investigate the potential clinical benefits of the described technique.

Press-fit techniques in ACLR surgery involve securing the graft in the bone tunnels without the use of additional fixation devices such as interference screws.^8^ They provide several potential advantages, including reduced hardware-related complications and elimination of potential stress risers.^8^^,^^10^ In a recent study, Tátrai et al.^7^ found that a patellar tendon graft with a press-fit fixation technique without any fixation device reduced the incidence of femoral tunnel widening. Additionally, Arnold et al.^12^ showed that implant-free press-fit fixation provided adequate primary stability. They commented that press-fit fixation was safe for clinical application.

Press-fit fixation also promotes biological healing and graft incorporation into bone, allowing for a more natural and stable knee joint.^8^^,^^12^ This eliminates the need for an interference screw and the many potential complications related to it, such as screw misplacement, potential impingement, and abrasion, along with radiologic artifacts on magnetic resonance imaging and computed tomography scans.^3^

The patellar tendon autograft is known for its robustness and strength, and it has been associated with a lower risk of graft failure, providing long-term reliability and reducing the likelihood of reinjury while allowing for efficient rehabilitation and a quicker return to sports owing to its favorable biomechanical properties and the ability to mimic the native ACL.^13^ Using press-fit fixation of both ends of the graft has been associated with early loss of graft tension and slippage, given that the technique relies on achieving a precise match between the graft and bone tunnel diameters and any deviation may lead to inadequate fixation.^8^^,^^12^ Another potential drawback is the increased difficulty in graft tensioning and positioning during the operation, given that the press-fit method may limit the surgeon’s ability to fine-tune these aspects. Our technique addresses these disadvantages by, first, using an adjustable fixation device for the femur, thus securing a fully filled and stable femoral tunnel end, and second, allowing for fine-tuning owing to the trapezoidal shape. The bony end of the graft can be hammered in according to the position and tension of the femoral end and, on achievement of the desired stability, can be safely trimmed at its distal end. A prospective clinical study is already under way to evaluate and investigate the possible advantages of this technique.

## Disclosures

All authors (Y.K., A.F., A.Y., A.E.P., K.Ö., E.V.) declare that they have no known competing financial interests or personal relationships that could have appeared to influence the work reported in this paper.

## Supplementary Data

Video 1 After clinical and radiologic confirmation of anterior cruciate ligament rupture, the patient is placed supine on the surgical table. The technique is shown applied on the left knee. The leg holder is removed from beneath the index extremity, and the knee is left floating in 90° of flexion. A longitudinal incision is performed, starting from the center of the patella down to the tibial tubercle. A ruler is then used to mark a tendon thickness of 10 mm from the middle of the patellar tendon. Superiorly, the patellar periosteum is marked with a length of 30 to 40 mm and a width of 10 mm. Inferiorly, the tibial tubercle is marked in a trapezoidal shape with a length of approximately 35 mm, a narrow superior base of 10 mm, and a wider inferior base of 12 mm. After graft extraction, the loop of an adjustable fixation device is brought in, and the periosteum is flipped over the loop and is clamped with the proximal part of the patellar tendon. Marks are placed over the tendon to designate the graft amount destined for the femoral tunnel. Starting from the 20-mm mark and working our way proximally, the tendon, the periosteum, and the loop of the adjustable fixation device are fixed with a double-needled reinforced loop suture in a whipstitch fashion. After 3 or 4 passages, the needles meet at the distal end of the patellar tendon graft and are shuttled first through the loop of the adjustable fixation device and then twice from lateral to medial through the graft. The suture ends are knotted.1
